# Analysis of the microglia transcriptome across the human lifespan using single cell RNA sequencing

**DOI:** 10.1186/s12974-023-02809-7

**Published:** 2023-05-30

**Authors:** Moein Yaqubi, Adam M. R. Groh, Marie-France Dorion, Elia Afanasiev, Julia Xiao Xuan Luo, Hadi Hashemi, Sarthak Sinha, Nicholas W. Kieran, Manon Blain, Qiao-Ling Cui, Jeff Biernaskie, Myriam Srour, Roy Dudley, Jeffery A. Hall, Joshua A. Sonnen, Nathalie Arbour, Alexandre Prat, Jo Anne Stratton, Jack Antel, Luke M. Healy

**Affiliations:** 1 Neuroimmunology Unit, Department of Neurology and Neurosurgery, Montreal Neurological Institute and Hospital, McGill University, Montreal, QC Canada; 2 Department of Microbiology and Immunology, Montreal Neurological Institute and Hospital, McGill University, Montreal, QC Canada; 3 Department of Electrical and Electronic Engineering, Shiraz University of Technology, Shiraz, Fars Iran; 4 Department of Comparative Biology and Experimental Medicine, University of Calgary, Calgary, AB Canada; 5 Department of Pediatric Neurosurgery, Montreal Children’s Hospital, Montreal, QC Canada; 6 Departments of Pathology, Neurology and Neurosurgery, McGill University, Montreal, QC Canada; 7 Neuroimmunology Research Laboratory, Centre de Recherche du Centre Hospitalier de L, Université de Montréal (CRCHUM), Montreal, QC Canada; 8 Department of Neurosciences, Université de Montréal, Montreal, QC Canada

**Keywords:** scRNA-seq, Ex vivo human microglia, Transcriptional heterogeneity, Gene regulatory network

## Abstract

**Background:**

Microglia are tissue resident macrophages with a wide range of critically important functions in central nervous system development and homeostasis.

**Method:**

In this study, we aimed to characterize the transcriptional landscape of ex vivo human microglia across different developmental ages using cells derived from pre-natal, pediatric, adolescent, and adult brain samples. We further confirmed our transcriptional observations using ELISA and RNAscope.

**Results:**

We showed that pre-natal microglia have a distinct transcriptional and regulatory signature relative to their post-natal counterparts that includes an upregulation of phagocytic pathways. We confirmed upregulation of *CD36*, a positive regulator of phagocytosis, in pre-natal samples compared to adult samples in situ. Moreover, we showed adult microglia have more pro-inflammatory signature compared to microglia from other developmental ages. We indicated that adult microglia are more immune responsive by secreting increased levels of pro-inflammatory cytokines in response to LPS treatment compared to the pre-natal microglia. We further validated in situ up-regulation of *IL18* and *CXCR4* in human adult brain section compared to the pre-natal brain section. Finally, trajectory analysis indicated that the transcriptional signatures adopted by microglia throughout development are in response to a changing brain microenvironment and do not reflect predetermined developmental states.

**Conclusion:**

In all, this study provides unique insight into the development of human microglia and a useful reference for understanding microglial contribution to developmental and age-related human disease.

**Supplementary Information:**

The online version contains supplementary material available at 10.1186/s12974-023-02809-7.

## Background

Microglia are central nervous system (CNS) resident myeloid cells that originate from early yolk sac progenitor cells [1]. Microglia seed the mouse brain at day 9 of embryonic life (E9.5) [1] and at an equivalent developmental timepoint of 5.5 gestational weeks in humans [2]. As they are implicated in the maintenance of homeostasis throughout the entire life span of both humans and mice [3], the disruption of homeostatic microglial functions during pre-natal and early post-natal development can adversely affect neural progenitors and lead to behavioral deficits in adulthood [4, 5]. Failure in neuronal network modulation by microglial pruning of synapses can contribute to an array of neurological disorders including epilepsy [6], schizophrenia [7] and autism spectrum disorder [8]. During early post-natal development, microglia have been shown to regulate myelinogenesis by altering oligodendrocyte precursor cell (OPC) survival through the production and release of insulin-like growth factor-1 (IGF1) [9]. The importance of microglia in the development of the pre- and post-natal brain is highlighted in cases of pediatric-onset leukoencephalopathy caused by homozygous mutations in colony stimulating factor 1 receptor (CSF1R), a tyrosine kinase critical for the development and survival of microglia [10]. As development continues beyond the post-natal stage, microglia acquire the innate immune function for which they are classically known. Genome wide association studies, transcriptomic analyses, and epigenomic studies all implicate microglia in the pathogenesis of adult-onset and age-related neurological diseases, such as Alzheimer’s disease [11], Parkinson’s disease [12] and Multiple Sclerosis [13]. Finally, heterozygous mutations in CSF1R cause adult-onset leukoencephalopathy with axonal spheroids and pigmented glia (ALSP), a disease characterized by neuropsychiatric presentation, seizures, confluent multifocal white matter abnormalities and widespread brain matter loss [14, 15]. Proper microglial function is therefore crucial for both early development and adult brain health.

Bulk and single-cell RNA-sequencing (scRNA-seq) have been used to describe the molecular diversity of microglia in both humans and mice at different developmental stages. Three waves of transcriptomic expression changes from pre-natal to adulthood have been described in the mouse brain [16]. Additional mouse studies corroborate the finding that microglia acquire distinct expression signatures at different time points of development and across various brain regions [17–19]. While these studies identified distinct maturation stages of microglia in mice, this may not accurately reflect equivalent processes in the human system [20, 21]. Based on a study of 1180 human adult cortical microglia, Masuda et al. reported 4 microglia clusters in the healthy human brain [19]. In a follow up study in which 4396 microglia were sequenced from individuals aged between 14 and 74 years, the same group identified 8 microglial-subtypes [22]. However, the majority of human studies to date have analyzed the microglial signature of pre-natal [23] and adult [19, 22] tissue samples but have neglected the pediatric and adolescent age range. Our understanding of microglia maturation throughout human development remains limited due to the inaccessibility of optimal brain material.

In the present study, we performed whole cell single cell-sequencing on 13,568 microglia isolated from surgical samples of 2nd trimester pre-natal, pediatric (18 months–2 years old), adolescent (10–14 years old) and adult (40–62 years old) brains. We show that (i) microglia maturation is completed by the 2nd trimester and the transcriptional states that microglia adopt are likely a response to the changing brain microenvironment rather than as a result of predetermined transcriptional programing, (ii) pre-natal microglia have a distinct transcriptomic expression profile compared to all post-natal microglia, including a gene signature that suggests a higher phagocytic capacity, (iii) post-natal microglia are more immune-responsive than pre-natal microglia, and (iv) pre- and post-natal microglia have distinct transcription factor activity profiles. Overall, this study provides a unique reference for the study of human microglia development and contributes to our understanding of microglial involvement in the maintenance of CNS homeostasis and their potential contribution to neurodegenerative diseases. To facilitate the use of our scRNA-seq datasets, we have also developed a straightforward online tool for evaluating gene expression at single-cell resolution (https://stratton-lab.github.io/dataviz) [24].

## Method

### Human brain samples

Post-surgery human brain tissues were obtained from the Department of Neuropathology at the Montreal Neurological Institute and Hospital (MNI) and the Montreal Children’s Hospital with written consent from families. Use of adult tissues were approved by the MNI Neurosciences Research Ethics Board (Protocol ANTJ 1988/3) whereas use of pediatric tissues was approved by the Montreal Children’s Hospital Research Ethics Board. Using a cavitron ultrasonic aspirator (CUSA), post-natal brain tissues were obtained from normal superficial tissue that had to be removed to get to the area of underlying pathology (i.e., the superficial surgical corridor on the way to the pathological tissue). Tissues from the normal appearing CUSA bag material and from pathologic site resected material were subjected to neuropathologic examination. Neuropathologic analysis of CUSA tissue samples confirmed that the fragments were comprised of histologically normal tissue. Second-trimester pre-natal human whole brain samples were obtained from the University of Washington Birth Defects Research Laboratory (MP-37-2014-540; 13-244-PED; eReviews_3345). Complete sample information is reported in Additional file 4: Table S1.

### Tissue processing and preparation of single cell suspensions

The detailed procedure of microglia isolation is explained in Durafourt et al. [25]. Briefly, post-natal brain samples were subjected to enzymatic 0.05% Trypsin (ThermoFisher) and 50 μg/ml DNAase (Roche) treatment for 15 min, and mechanical dissociation was done by passing the tissue through a nylon mesh, once brought to the laboratory. The resulting cell suspensions were subjected to Percoll gradients to remove myelin. Total cell populations for each post-natal sample (mainly composed of microglia and oligodendrocyte lineage cells) were then used for single cell RNA-sequencing and library preparation. Pre-natal samples also underwent enzymatic and mechanical dissociation; however, because there is no myelin at the pre-natal time point, the Percoll gradient step was skipped and the total cell population was sent directly for scRNA-seq.

### Fluorescent activated cell sorting (FACS)

One of the pre-natal samples was subjected to FACS. For this sample, following enzymatic and mechanical dissociation, the cells were resuspended in FACS buffer containing CD11b and CD45 antibodies. Samples were kept on ice and sorted by a BD FACSAria Solution. The resulting sorted cells were used for single cell RNA-sequencing.

### Single cell RNA-sequencing data analysis

All samples were sequenced at McGill University and the Génome Québec Centre. 10X Chromium v2.0 technology was used for cell single cell capturing and library preparation. scRNA-seq was performed on the Illumina HiSeq4000 PE75 sequencer. The 10XGenomics CellRanger pipeline was used to demultiplex the cells and their unique molecular identifier barcodes, and to align reads to the GRCh38 human reference genome. The subsequent analyses were completed using the Seurat (v3.1) R package [26].

Individual datasets were subjected to standard Seurat pipeline for quality control, gene expression normalization, batch-effect correction, clustering, and differential expression analysis. Briefly, any cell with a genome that was comprised of 5 to 12 percent mitochondrial genes was considered a dead cell and was removed from analysis. Similarly, any cell that contained less than 200 or more than 2500 unique feature counts was considered a low-quality cell and was removed from the downstream analysis. Moreover, genes that have been shown to be artefacts of cell isolation procedures were deleted from the gene-barcode matrix [22, 27]. Expression of the sorted pre-natal sample was normalized according to expression of housekeeping genes from the non-sorted prenatal samples. After these quality control steps, gene expression levels of the cells were natural log normalized and scaled. In order to overcome single cell RNA sequencing drop-out, we limited our analysis to a subset of 2000 highly variable genes for each dataset. Principle component analysis (PCA) was performed on highly variable genes to reduce the dimensionality of the data. The first 20 principle components were selected for clustering. A shared-nearest neighbor graph was constructed based on PCA analysis and the Louvain clustering algorithm was used several times to identify clusters at multiple resolutions. Individual datasets were analyzed using a resolution of 0.6. Finally, the uniform manifold approximation and projection (UMAP) algorithm was used to visualize the clusters in two-dimensional space. The expression levels of cell-type marker genes were used to determine the identity of each cluster. Microglia from each individual dataset were subset using the expression of *TREM2* and *C1QA*. Microglia selected from each individual dataset were subjected to dataset integration as follows: Following down sampling of post-natal microglia according to the number of pre-natal cells (3500 cells per developmental stage), all datasets were integrated using the Seurat “FindIntegrationAnchors” and “IntegrateData” functions, which perform canonical correlation analysis (CCA) to remove any possible batch effects between datasets. The integrated object was then re-clustered. For PCA plotting, cell cycle related genes were regressed out using the Seurat cell-cycle scoring and regression pipeline. The optimal resolution for clustering was determined using the “clustree” R package [28]. This package measures the stability of clusters at different resolutions, and a high degree of movement between clusters is considered over clustering. We chose a resolution of 1 for our clustering purposes. Differentially expressed genes were identified using the “FindMarkers” function in Seurat based on Wilcox test scores for genes that were detected in at least 25% of cells in the populations compared. Heatmap plots of the top marker genes were generated using the DoHeatmap function. The top 100 highly variable genes per developmental stage for gene ontology assessment were obtained by calculating the variance of each group versus the other three groups at a time and selecting the top 100 highly expressed genes.

### Pseudotime trajectory inference

Pseudotime trajectory inference analysis was done using the PhateR (v1.0) package to order cells in “pseudotime” and visually highlight transitions between cells using dimensional reduction methods that minimize gene expression differences between sequential cell pairs. PhateR uses the “potential of heat diffusion for affinity-based embedding” (PHATE) method created by Moon et al. [29]. Briefly, PHATE encodes local data via local similarities, encodes global relationships using potential distances based on diffusion probabilities, and embeds potential distance information into low dimensions via metric multi-dimensional scaling [29]. The phate algorithm was run on the integrated counts derived from the Seurat object with the gamma parameter set to gamma = 0 and the remaining parameters set to default (knn = 5, decay = 40, optimal t was automatically selected to be *t* = 8). PhateR was chosen for the trajectory inference analysis as it does not rely on prior assumptions of data structure.

### Mouse single cell RNAseq data analysis

Raw expression matrices of 12 single cell RNA-sequencing samples were downloaded from Gene Expression Omnibus (GEO) with the accession number 121654 [17]. Each dataset was subjected to Seurat analysis in the same way as the human samples. The average expression of genes from each developmental stage was measured using the “AverageExpression” function in Seurat.

### Jaccard similarity matrix

The weighted Jaccard similarity index calculates the similarity between two or more numeric vectors. In our study, we used a Jaccard similarity matrix to measure the similarity between the average expression of genes between developmental stages. The average expression levels of genes for the whole population of microglia at each developmental stage were calculated using the “AverageExpression” function in Seurat. The range of this similarity is from 0 to 1, in which 1 is highest similarity and 0 is lowest similarity between samples. The Jaccard similarity index is calculated using the sum of minimum values in two vectors at a time (the intersection of two RNA-seq data samples) divided by the sum of the maximum values in both vectors (the union of each). We used MATLAB to calculate the Jaccard similarity index automatically.

### Gene regulatory network analysis

Single cell regulatory network inference and clustering (SCENIC) [30] was employed to infer transcription factor (TF) activity by identifying target genes that were co‐expressed with TFs using a random forest model. Analysis was performed using default and recommended parameters as suggested on the SCENIC vignette (https://github.com/aertslab/SCENIC) using the hg19 mc9nr (Version 9) RcisTarget database (https://resources.aertslab.org/cistarget). Kernel density histograms that plotted the differential AUC distribution across microglia of different developmental stages and the targetome of individual TFs were queried using previously described modifications [31]. Briefly, histograms were plotted using the regulon activity matrix (‘3.4_regulonAUC.Rds’), in which columns represent cells and rows represent the AUC regulon activity and the targetome of individual TFs, both which were queried using the regulon data frame (‘2.6_regulons_asGeneSet.Rds’).

### PCA analysis for pseudo-bulk analysis and batch effect correction

The average expression of microglia genes from each developmental stage was measured using the “AverageExpression” function in Seurat. Following batch effect correction, these values were used to measure the principal component scores using the built-in function of R called prcomp, and the results were visualized using ggplot2 in R. The batch effect between human and mouse samples was removed using the ComBat function in the SVA package using default parameters [32].

### Pearson correlation

Pearson correlation analysis was employed to check the similarity of the human and mouse samples. The correlation coefficient was measured and visualized using MATLAB’s built-in functions.

### Gene ontology analysis

Gene ontology analysis was performed using the gProfiler web tool. Only significant terms with adjusted *p*-value < 0.05 were presented.

### In situ RNA hybridization

Healthy adult human brain tissue was sourced from the laboratory of Dr. Alexandre Prat. Pre-natal human brain tissue was sourced from the laboratory of Dr. Nathalie Arbour. The in situ hybridization procedure was completed as per the Advanced Cell Diagnostics (ACD) RNAscope^®^ Multiplex Fluorescent v2 protocol for fresh-frozen tissue, unless otherwise stated. In brief, both adult and pre-natal fresh-frozen slides (10-15uM sections) were removed from − 80 °C storage where they were sealed in aluminum foil, thawed, and immediately fixed on-slide at 4 °C in pre-chilled 4% PFA for 15 min. Slides were then rinsed in PBS, dehydrated with xylene and alcohol at RT, and a hydrophobic barrier was applied (ImmEdge, Vector Labs; Cat. No.310018). Once completely dry, the slides were incubated with protease IV at RT for 30 min; the timing of this step was critical and may require optimization depending on the tissue type used. The remainder of the protocol was completed as per ACD reccomendations, including probe, Multiplex FL v2 AMP and HRP, and Opal™ fluorophore incubations, all at 40 °C using the ACD HybEZTM Humidifying System/RNAscope^®^ EZ-Batch™ Slide Holder and Wash Tray. Washes were completed with RNAscope^®^ wash buffer (Cat. No. 310091), and may also need to be adjusted to minimize tissue erosion. The probes used were AIF1 (Cat. No. 433121), CXCR4 (Cat. No. 310511), IL18 (Cat. No. 400301) and CD36 (Cat. No. 536631). The OpalTM fluorophores used were 520, 570, 620, and 690, with Cat. Nos. FP1487001KT, FP1488001KT, FP1495001KT and FP1497001KT, respectively. Fluorophores were diluted in TSA buffer (1:1500; Cat. No. 322809). Nuclei were stained with ACD-supplied DAPI for 30 s at RT and slides were mounted using ProLongTM Gold Antifade Mountant (ThermoFisher, Cat. No. P36930).

Slides were imaged using a Zeiss Axio Observer at 20× for representative images, and 40× for corrected total cell fluorescence (CTCF) quantification. In short, CTCF values were generated by manually segmenting microglia (at least 20 cells) three separate times to obtain average total microglial area, mean intensity, and integrated density. Adjacent background selections of the same area as each combination of selected microglia were also taken simultaneously for comparison. These values were then input into the corrected total cell fluorescence equation to generate individual CTCF metrics: CTCF = Integrated density—(area of selected cell * mean fluorescence of background readings). CTCF values were then plotted using RStudio (2022.07.0).

### Cell culture

Pre-natal and post-natal microglia were cultured in DMEM (Sigma) with 5% fetal bovine serum (FBS; Wisent BioProducts) + 1% penicillin/streptomycin (P/S; Gibco) and MEM (Sigma) with 5% FBS + 1% P/S + 1% GlutaMAX (Gibco) + 0.1% glucose (Sigma), respectively. Cells were maintained at 37 °C under a 5% CO_2_ atmosphere.

### ELISA

Supernatants of pre-natal and adult microglia treated with LPS (100 ng/ml) for 24 h were retrieved. ELISA for TNF and IL-6 was performed in duplicate following the manufacturer’s instructions (BD Biosciences).

### Myelin uptake assay

Human myelin debris were obtained and labelled with pHRodo Green STP Ester (Invitrogen) as previously described (Healy et al. [43]). Cells were plated in a black 96-well plate at equal density and challenged with 15 ng/mL of myelin debris. Following the incubation with myelin, cells were counterstained with Hoechst 33,342 (5 g/mL, Invitrogen) and green fluorescence intensity per cell was measured using a CellInsight CX7 High Content Screening Platform. All conditions were assessed in triplicate. GraphPad Prism 8.0 software was used for non linear regression of the kinetic curve, t50 estimation and t-test analysis. A *p*-value lower than 0.05 was considered statistically significant.

## Results

### Isolation and characterization of human microglia

**Fig. 1:**
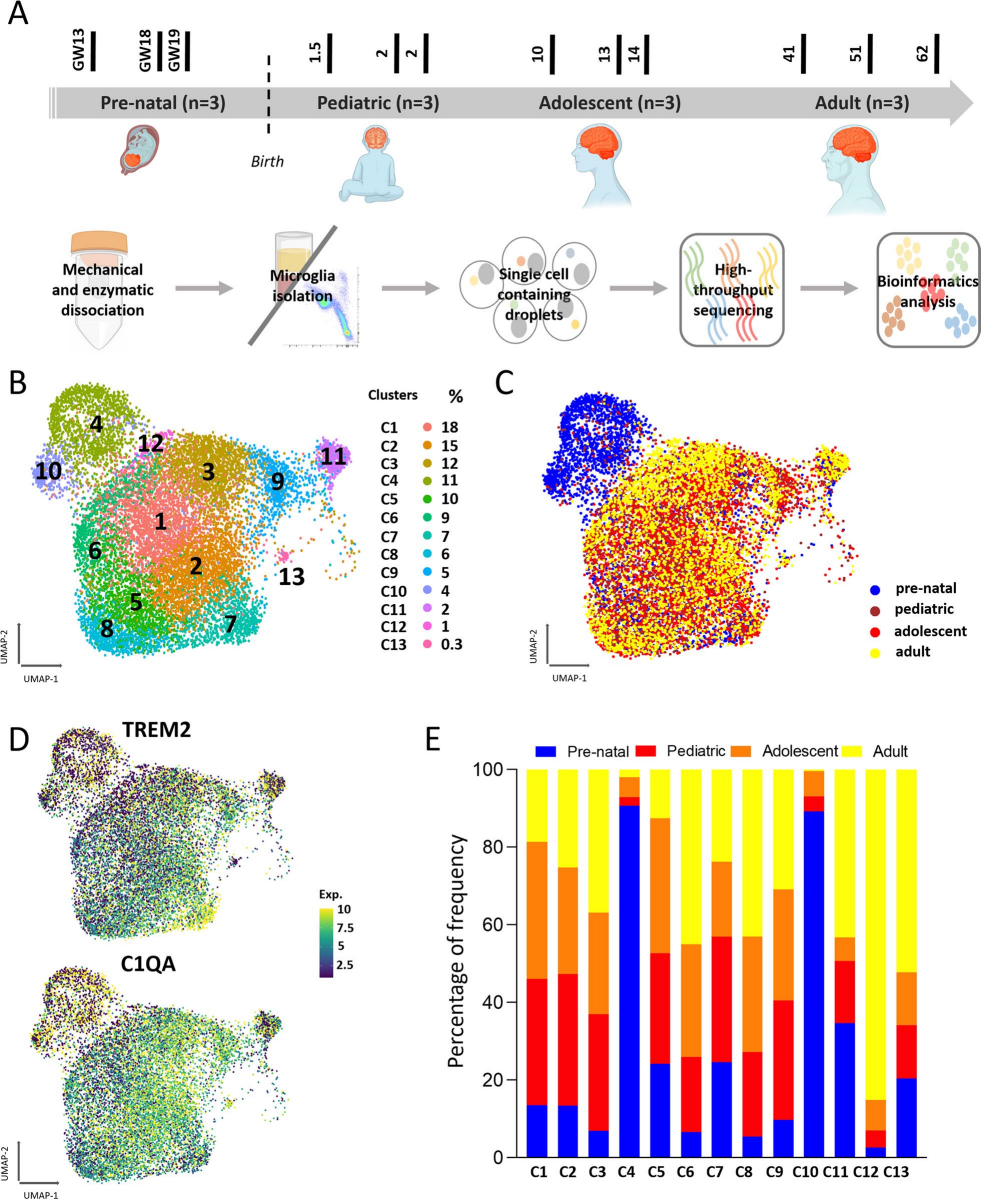
Isolation and characterization of human microglia. **A** Microglia were isolated from brain tissues at different ages, followed by tissue processing, single cell RNA sequencing and bioinformatics analysis. Three biological replicates per developmental stage (*n* = 12 in total) were used for downstream analysis. **B** UMAP plot of 13 clusters of 13,583 isolated microglia and relative size of each cluster. **C** UMAP plot representing the distinct expression profile of pre- compared to post-natal cells. Each age is represented by a separate color. **D** UMAP plot showing normalized expression of canonical microglia genes in all cells. **E** Stack bar plot showing the contribution of microglia from each age in each cluster. Different ages are represented by a distinct color

To study changes in the transcriptional profile of microglia over time, human brain samples were collected at different developmental stages. Pre-natal samples were obtained from second trimester pre-natal brain tissue (Fig. 1A, Additional file 4: Table S1). Post-natal brain samples were obtained from patients undergoing surgical resection of CNS tissue. The collected tissues were obtained from normal appearing, superficial tissue, removed to access underlying pathology (i.e., the superficial surgical corridor distal to the site of pathology). Post-natal samples were collected from individuals grouped into three age brackets: pediatric (1.5–2 years), adolescent (10–14 years) and adult (41–61 years) (Fig. 1A, Additional file 4: Table S1).

Post-natal tissue was enzymatically and mechanically digested, followed by cell separation using a Percoll gradient. The cell suspension was subjected to scRNA-seq using droplet-based sequencing through a 10× platform (Fig. 1A). Isolated cells were predominantly microglia and oligodendrocytes, with minor populations of border associated macrophages (BAM), OPCs, astrocytes and lymphocytes (Additional file 1: Fig. S1A) according to the expression of canonical markers of each cell type [24, 33]. Microglia were selected for further analysis based on the expression of canonical microglia markers complement c1q A chain (*C1QA*) and triggering receptor expressed on myeloid cells 2 (*TREM2*) (Additional file 1: Fig. S1B). Following removal of low-quality cells, 14,520 post-natal microglia were retained for downstream analysis. Pre-natal samples were enzymatically and mechanically digested; due to the absence of myelin in the pre-natal brain, a Percoll separation was not carried out. Of the three pre-natal samples obtained, two were processed as above and one sample was subjected to fluorescence-activated cell sorting (FACS) (live, CD45 + /CD11B +) ((Additional file 1: Fig. S1C). In total 4,211 pre-natal microglia expressing canonical microglia markers of *TREM2* and *C1QA* were obtained (Additional file 1: Fig. S1D). Prior to downstream analysis, a population of highly proliferative microglia expressing proliferation markers cyclin dependent kinase (*CDK1*) and DNA topoisomerase II alpha (*TOP2A*) were excluded (Additional file 1: Fig. S1D) to avoid the over-representation of proliferation associated genes in the differential expression analysis between pre- and post-natal cells.

Following quality control checks and removal of the proliferative cluster, 3400 pre-natal microglia were retained for further analysis. The post-natal microglia population was down-sampled to match that of the pre-natal samples. The down-sampling was done by randomly selecting cells from each developmental stage to make sure the observed result was not skewed by differing cell numbers in each dataset. Following integration of pre- and post-natal microglia populations, 13,583 cells with an average of 91,879 reads and 1234 genes per cell were obtained.

### Microglia transcriptional clusters link to specific functions

We next aimed to determine the extent of microglia transcriptional heterogeneity at different timepoints in human development. To do this, we integrated all cells and performed unsupervised clustering, generating 13 clusters of varied sizes (Fig. 1B) grouped into two primary clouds. One cloud was composed primarily of pre-natal microglia and the other post-natal microglia (Fig. 1C, Additional file 1: Fig. S1E). Analysis revealed a continuum of gene expression between post-natal developmental stages, resulting in diffuse localization as opposed to compact clustering within the larger cloud of cells (Fig. 1C). Additionally, we confirmed that all cells from all clusters expressed canonical microglia markers including *C1QA*, *TREM2, CX3CR1, GRP34, GPR183, P2RY12, P2RY13 and ADCRG1* (Fig. 1D, Additional file 1: Fig. S1F). Our analysis showed that each age group made differential contribution to each cluster (Fig. 1E). For instance, clusters 4 and 10 were predominantly comprised of pre-natal cells, cluster 12 predominantly adult cells, and cells from each age contributed in relatively equal proportion to cluster 2 (Fig. 1E).

**Fig. 2:**
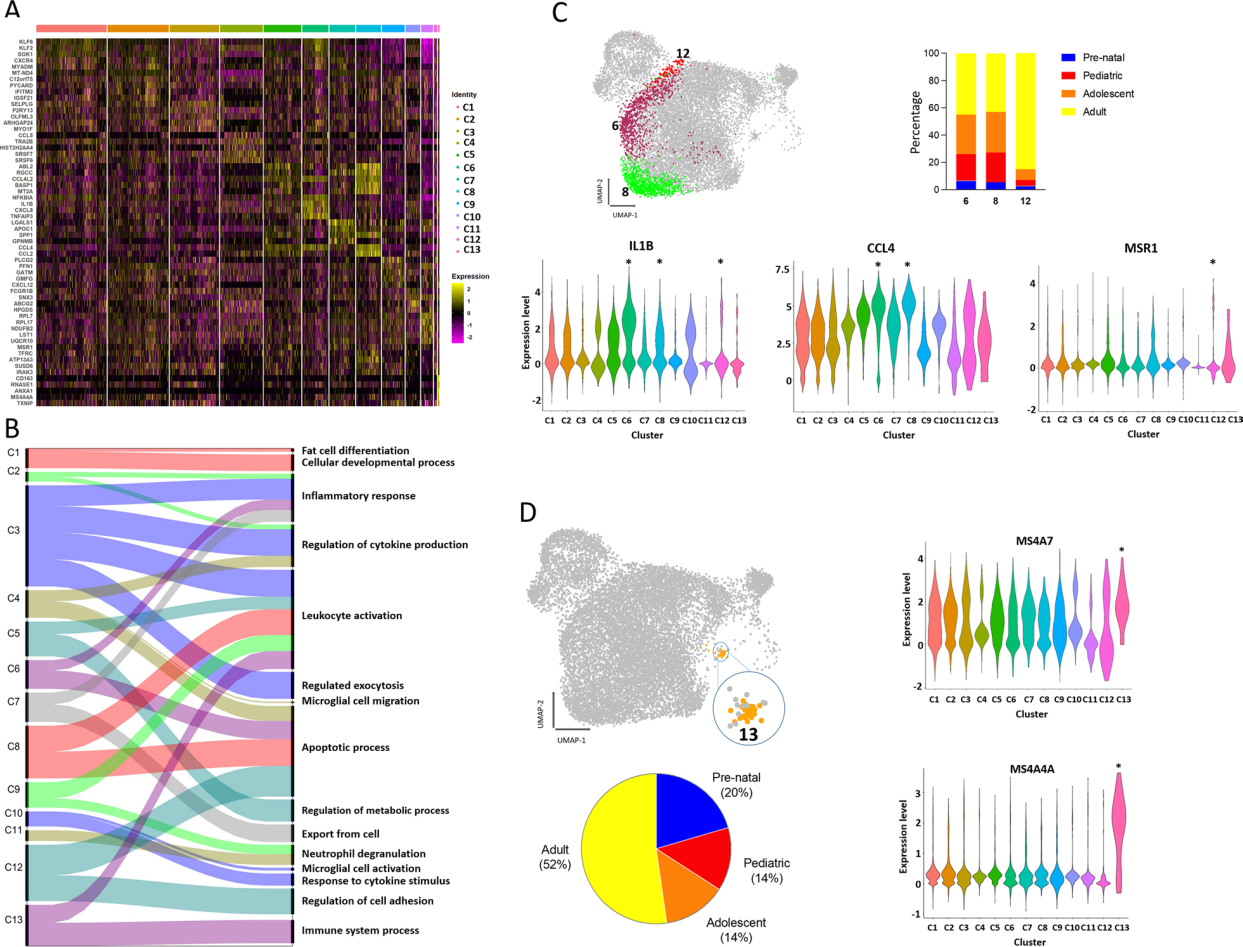
Microglia transcriptional clusters link to specific functions. **A** Heatmap of top 10 DEGs per cluster. Gene expression is represented by a color-coded z-score. Up and down regulated genes are shown by yellow and violet colors respectively. **B** Alluvial plot representing the most affected biological processes by upregulated genes of each cluster. Ribbon thickness indicates the number of genes per biological process. **C** UMAP plot highlighting three microglia clusters associated with inflammatory phenotypes. Stack bar plot showing contribution of each age group to these three clusters. Each age group is represented by a distinct color. Violin plot depicting expression of significantly upregulated gene in these three clusters *adjusted *p*-value < 0.05 (Wilcox test). **D** UMAP plot highlighting microglia originating from the second wave of microglia development. Pie chart showing the contribution of microglia of each age to cluster 13. Each age is represented by a distinct color. Violin plots showing expression of *MS4A4A* and *MS4A7* genes * adjusted *p*-value < 0.05 (Wilcox test)

Hierarchal clustering using the top differentially expressed genes (DEGs) between each cluster revealed different transcriptomic signatures (Fig. 2A, Additional file 5: Table S2). Certain microglia clusters were associated with specific functions (Fig. 2B). Clusters 6, 8, and 12, constituted of post-natal microglia (specifically adult microglia), expressing well known mediators of the inflammatory response such as interleukin 1 beta (*IL1B*) and macrophage scavenger receptor 1 (*MSR1*) (Fig. 2C). The most upregulated gene of cluster 8 was C–C motif chemokine ligand 4 (*CCL4*) which is involved in the recruitment of peripheral immune cells to the CNS (Fig. 2C). Previous studies using scRNA-seq and single molecule fluorescence in situ hybridization (smFish) have confirmed the presence of *Ccl4* + microglia in mice [17, 34]. The most significant biological processes associated with these three clusters were inflammatory processes, leukocyte activation, and apoptotic processes (Fig. 2B). The upregulation of inflammation-associated pathways and of cytokine expression in adult cells might suggest that they are chronically activated or that they have heightened readiness to respond to insults.

In cluster 13 which contains a majority population of adult microglia (52%) we found an upregulation of genes belonging to the membrane-spanning 4A (*MS4A*) family, specifically *MS4A4A* and *MS4A7*, commonly considered to be macrophage markers (Fig. 2D) [33]. Although expression of these two genes is not exclusive to adult cells, their expression level was higher within this cluster in comparison to cells of other ages (Additional file 1: Fig. S1G). These cells might be representing a group of cells that have been generated through a second wave of microglia development along side canonical microglia developmental pathway.

Altogether, our data indicate that the major transcriptomic difference between human microglia of different ages is between that of pre- and post-natal cells. Further, we show that there are subtle yet potentially significant transcriptomic differences in microglia at different stages of post-natal life, including the upregulation of inflammatory pathways in adult microglia.

### Age-associated gene expression signatures of human microglia

**Fig. 3:**
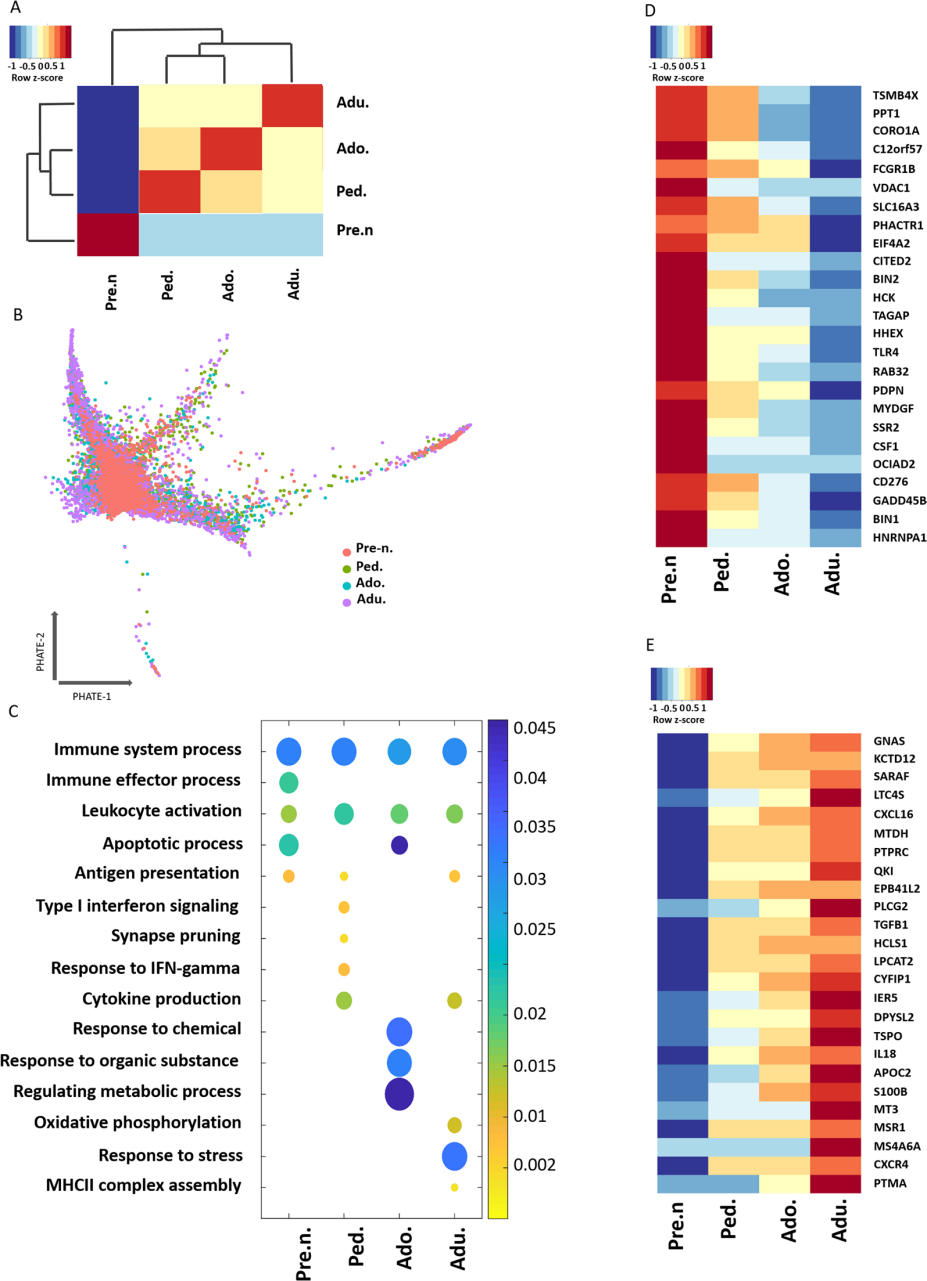
Transcriptional landscape of microglia at distinct developmental ages. **A** Hierarchal clustering of microglia at each age according to Jaccard similarity coefficient score which is represented by a color gradient, yellow (highest similarity) and dark blue (lowest similarity). **B** PHATE visualization for ex vivo human microglia by age. **C** Dot plot depicting the most affected biological processes of the top 100 highly variable genes for each age. The circle size indicates the number of genes present in each biological process. Statistical significance of terms is represented by color gradient, *p*-value of all terms is < 0.05. **D**, **E** Heatmap showing expression of top 25 highly variable genes whose expression incrementally decreased **D** or increased **E** with age. Red and blue color indicate up and down regulation of genes, respectively

**Fig. 4:**
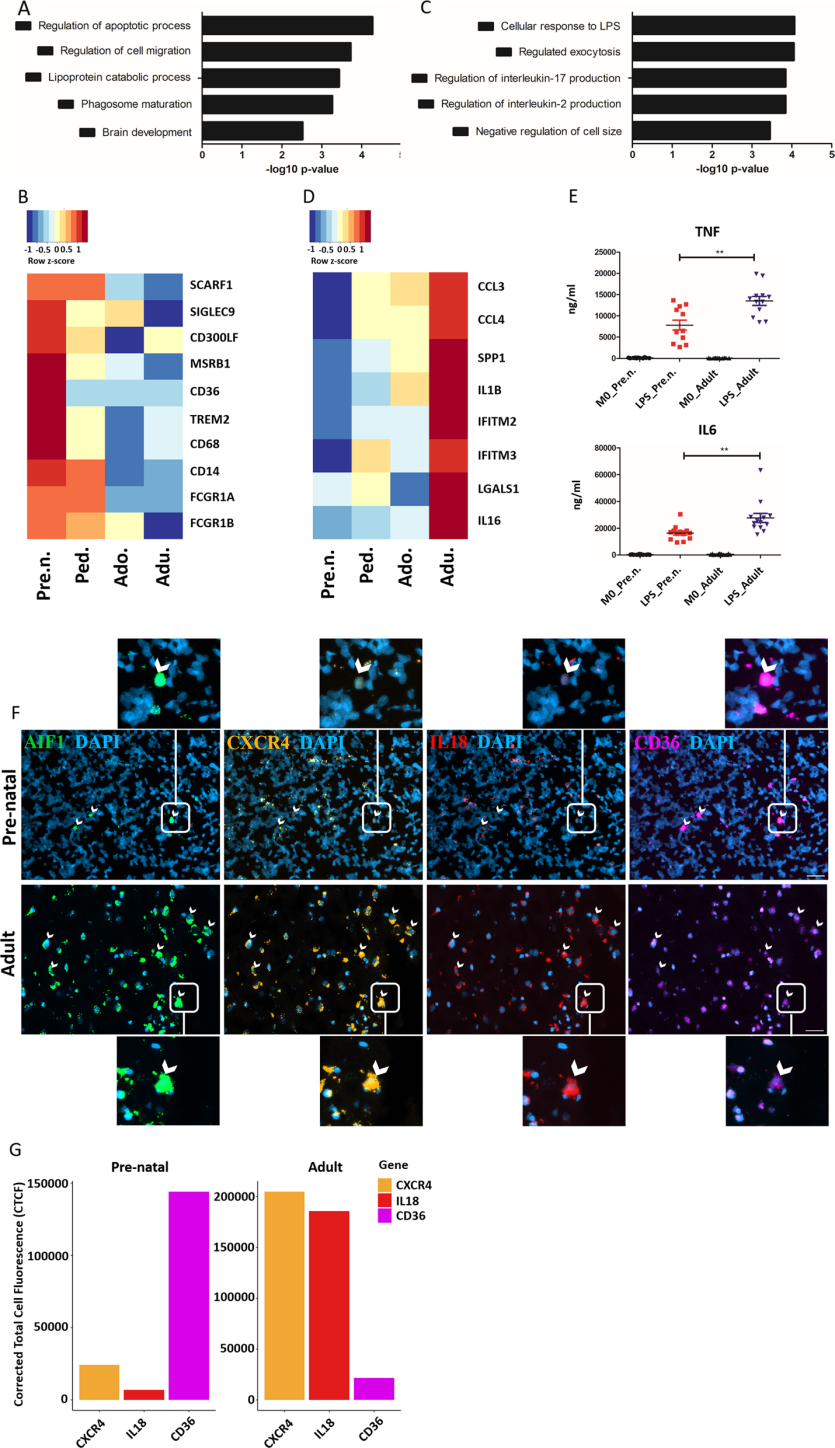
Age-associated gene expression signatures of human microglia. **A** Gene ontology analysis of top 100 highly variable genes whose expression incrementally decreased with age. **B** Heatmap depicting expression of phagocytosis related genes. Red and blue color indicate up and down regulation of genes, respectively. **C** Gene ontology analysis of top 100 highly variable genes whose expression incrementally increased with age. **D** Heatmap depicting expression of pro-inflammatory molecules. Red and blue color indicate up and down regulation of genes, respectively. **E** Human pre-natal and adult microglia were treated with LPS 100 ng/ml for 24 h. Release of TNF and IL-6 was measured by ELISA. Wilcoxon–Mann–Whitney *t* test was used to check significance of the result. **F** Representative RNAscope images of *AIF1* (green), *CXCR4* (orange), *IL18* (red), and *CD36* (magenta) expression in fresh-frozen pre-natal and adult human brain tissue (*n* = 1 pre-natal; *n* = 1 adult). The absolute number of microglia is vastly reduced in pre-natal brain tissue compared to adult brain tissue, as visualized by *AIF1* expression. A selection of microglia is denoted by white arrowheads for ease of interpretation. Scale bar = 40 μM Enlarged images of individual cells expressing markers of interest are included and their position indicated by white boxes. **G** Bar plot of corrected total cell fluorescence (CTCF; a standardized intensity measurement) values for *CXCR4, IL18* and *CD36* for *AIF1* + cells*. CXCR4* and *IL18* expression are increased in adult microglia compared to pre-natal microglia. Conversely, *CD36* expression in pre-natal microglia far exceeds that of adult microglia

To further investigate differences between each developmental stage we grouped samples by their age group and averaged the gene expression level of each sample in the groups (Additional file 5: Table S3). Next, we generated a Jaccard similarity matrix to measure the gene expression similarity at each age using the full transcriptome content of the cells. The Jaccard similarity index ranges from 0 to 1, with 0 representing the lowest and 1 the greatest similarity. The analysis showed that pre-natal microglia were least similar to all other age groups (Fig. 3A). Among the post-natal stages, adolescent microglia were the least similar compared to adult and pediatric cells, although the correlation coefficients of all post-natal samples were above 0.8 (high) (Fig. 3A). Pseudotime trajectory analysis using Potential of Heat-diffusion for Affinity-based Trajectory Embedding (PHATE) indicated that microglia did not align on the trajectory plot according to their developmental age. Instead, microglia from each age group can be found distributed evenly across the pseudotime (Fig. 3B), suggesting the observed transcriptional differences between the cells are more functional in nature than developmental. Gene ontology analysis of the top 100 most highly expressed genes within each age group (Additional file 5: Table S4) identified immune system processes, leukocyte activation, and antigen processing and presentation as enriched pathways in all developmental stages (Fig. 3C). This data suggests that different genes regulate those same pathways during each developmental timeframe. Of the pathways uniquely upregulated at specific developmental stages, we noted upregulation of stress response genes in adult cells, (Fig. 3C), which may relate to their potentially higher inflammatory activities identified in Fig. 2C. In adolescent cells, there was an enrichment in the expression of metabolic genes, suggesting a greater metabolic activity of microglia or a shift in the metabolic state of the cells at this developmental stage (Fig. 3C). Pediatric microglia expressed high levels of genes involved in synaptic pruning, which is a prominent function of microglia at this time of development (Fig. 3C). Finally, even though apoptotic pathways were among the most upregulated processes in pre-natal and adolescent cells compared to other post-natal samples, we observed a significant enrichment of this term in the pre-natal cells (Fig. 3C). We next aimed to identify genes whose expression may correlate with the acquisition of a mature microglia phenotype. We identified two groups of genes, those whose expression decreased with age (Fig. 3D) and those whose expression increased with age (Fig. 3E). Gene ontology analysis of the top 100 most highly variable genes that were highest during embryogenesis and decreased over time (Additional file 5: Table S5) revealed an enrichment in genes involved in cell apoptosis regulation, phagocytosis, and brain development (Fig. 4A). A wide array of phagocytosis related genes were identified to be more highly expressed in pre-natal samples compared to post-natal samples (Fig. 4B). Higher expression of *CD36, TREM2* and *FCGR1B*, all positive regulators of phagocytosis, suggests that human pre-natal microglia may have greater phagocytic capacity than post-natal cells.

Gene ontology analysis of the top 100 most highly variable genes that were lowest during embryogenesis and increased over time (Additional file 5: Table S5) showed that these genes are related to positive regulation of IL-17 and IL-2 (Fig. 4C). The IL-17 signaling pathway is involved in the pathogenesis of chronic inflammatory and autoimmune diseases [36]. Several interferon-responsive genes (*CCL3*, *CCL4*, *IFITM2* and *IFITM3*) were upregulated in adult microglia as opposed to other ages (Fig. 4D). *SPP1*, the gene encoding osteopontin that has been previously associated with an aged microglia signature [37], was also upregulated in adult cells (Fig. 4D). Moreover, an ELISA experiment confirmed that adult microglia secrete higher levels of pro-inflammatory cytokines such as *TNF* and *IL6* in response to lipopolysaccharide (LPS) treatment as compared to pre-natal microglia (Fig. 4E).

Morepver, we performed validation experiments to confirm our transcriptional findings of *IL18*, *CXCR4* and *CD36* using RNAscope technology on healthy adult human autopsy and pre-natal human brain tissues (Fig. 4F and G). In line with our transcriptional observations, *IL18* and *CXCR4* showed significantly increased expression intensity in the adult section compared to the pre-natal section. In contrast, *CD36* showed increased expression intensity in the pre-natal section compared to the adult section (Fig. 4F and G).

Finally, in order to check greater phagocytic efficiency of pre-natal cells we conducted phagocytic activities using pHrodo Green-labelled human myelin. Myelin debris labelled with a pH indicator dye is a useful tool for the study of myelin phagocytosis (Healy et al. [43]). Uptake of pHRodo Green-labelled myelin debris was evaluated in pre-natal and post-natal microglia in culture over a time course of 15 min, 30 min and 2 h. The amount of time needed to reach half of the maximal phagocytic capacity of the cells was then estimated (t50) as an indicator of the speed of the phagocytic process (Additional file 2: Fig. S2A, B). Pre-natal microglia showed slightly faster phagocytosis of myelin debris, but this did not reach statistical significance (Additional file 2: Fig. S2A).

Overall, we found that human pre-natal microglia express a signature that indicates greater phagocytic capacity, while post-natal microglia appeared to be more immune-responsive. Finally, microglia from adolescent brains were more metabolically active than microglia at other developmental stages.

### Pre-natal microglia have a distinct transcription factor activity profile compared to post-natal microglia

**Fig. 5:**
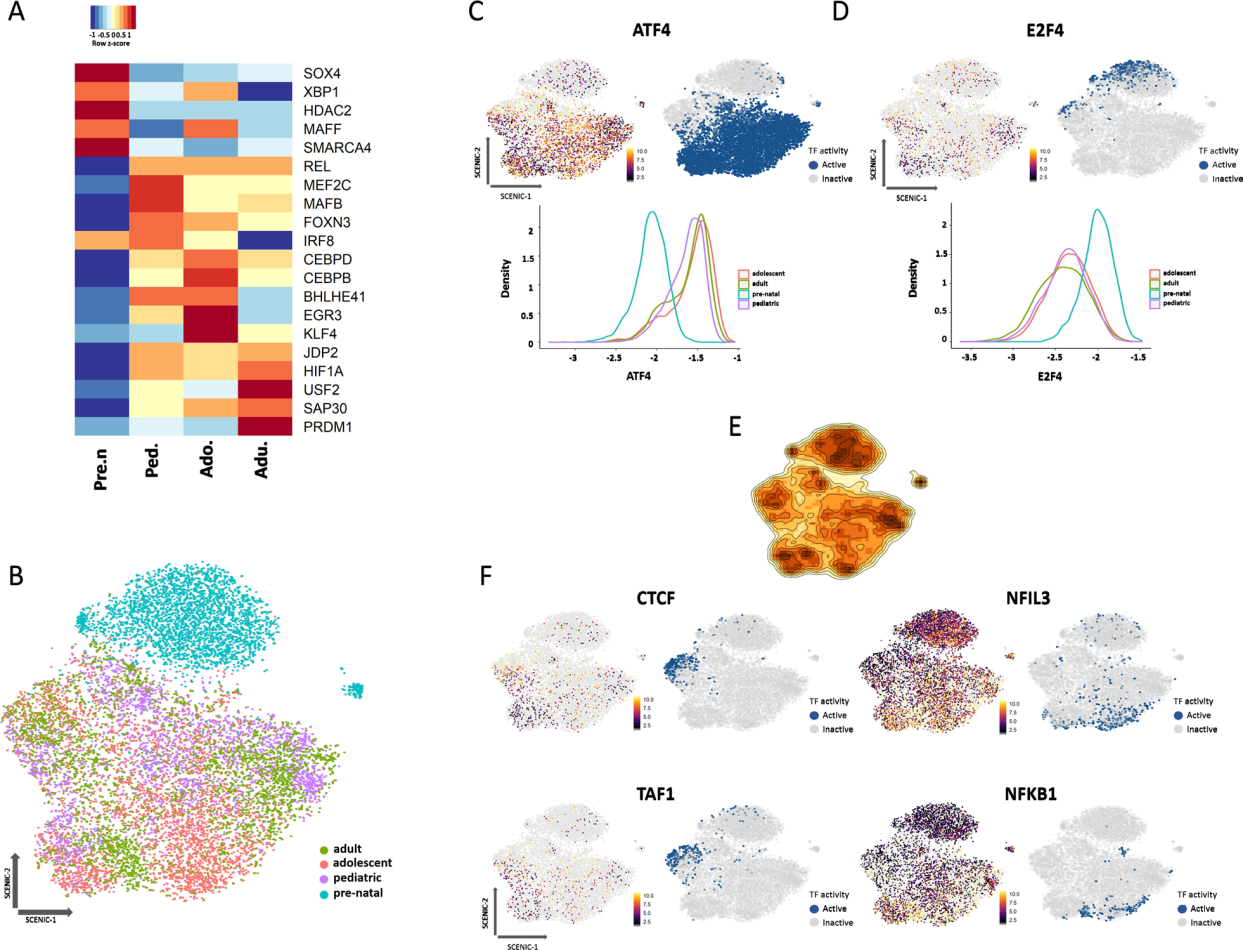
Pre-natal microglia have a distinct transcription factor activity profile compared to post-natal microglia. **A** Heatmap depicting top 5 microglia transcription factor signatures for each age group. Red and blue colors indicate up and down regulation of genes, respectively. **B** SCENIC plot depicting unbiased microglia clustering according to inferred TF activity separated by age. Each age group is represented by a distinct color. **C**, **D** SCENIC plot representing mRNA expression, binary regulon activity, and kernel density AUC histogram for *ATF4* and *E2F4* TF. Expression of genes is represented by a color gradient. TF activity is represented by a binary index in which blue denotes active and grey denotes inactive. **E** Density plot showing global activity map of TFs. Highly active and less active spots are represented by dark and light orange color respectively. **F** SCENIC plot representing mRNA expression and binary regulon activity of selected TFs. Expression of genes is represented by a color gradient. TF activity is represented by a binary index in which blue denotes active and grey denotes inactive

Transcription factors (TFs) are the main regulators of developmental and cellular transitions, as they control the expression of key downstream target genes. TFs and their downstream targeted genes make up what is known as a gene regulatory network (GRN). Single cell regulatory network inference and clustering (SCENIC) was utilized to identify the TFs relevant to gene expression regulation of microglia at each age. The SCENIC pipeline reconstructs GRNs by identifying gene sets co-expressed with TFs and by filtering out modules that exhibit low TF motif enrichment around or upstream of 5' transcription start sites [30]. SCENIC analysis provides a measurement of activity for a given TF. We measured both the transcriptional expression level of TFs in our transcriptomic dataset and the inferred activity of these TFs using SCENIC analysis and identified 142 TFs that potentially govern the activity of GRNs involved in microglia maturation. We found that certain TFs were uniquely upregulated in individual developmental stages compared to all others (Fig. 5A). The most expressed TFs for each age group compared to others were SRY-box transcription factor 4 (*SOX4*) for pre-natal microglia, V-maf musculoaponeurotic fibrosarcoma oncogene homolog B (*MAFB*) for pediatric microglia, CCAAT enhancer binding protein delta (*CEBPD*) for adolescent microglia and PR domain zinc finger protein 1 (*PRDM1*) for adult microglia (Fig. 5A). As expression differences alone do not provide insight into TF activity, we next examined the activity level of the previously identified 142 TFs. SCENIC analysis similarly identified TFs with higher activity at each developmental stage, with the most significant differences found between pre- and post-natal cells (Fig. 5B). Since TFs are sensitive to small changes of expression, clustering of the cells based on TF activity results in increased noise in the data (Additional file 3: Fig. S3A). Therefore, we considered a broader view of TF expression by considering each developmental stage (Fig. 5B). Interestingly, the activity of most TFs was higher in pre- than post-natal cells, with TF activities found to be similar among post-natal microglia (Additional file 3: Fig. S3B). Of the TFs identified that could distinguish pre- and post-natal microglia, some had functions associated with pathways previously identified to be upregulated at specific ages. For example, activating transcription factor 4 (*ATF4)*, which has been shown to regulate the inflammatory signature of microglia [38], displayed higher activity in post- versus pre-natal microglia (Fig. 5C). TFs such as E2F transcription factor 4 (*E2F4)*, a cell cycle regulator, had higher activity in pre-natal microglia, indicating a higher proliferative capacity versus post-natal cells (Fig. 5D). In addition, *SP1* was another TF whose activity found to be higher in pre-natal versus post-natal microglia (Additional file 3: Fig. S3C). Among the post-natal microglia, we identified ‘hotspots’ of specific TF activity regulating gene expression in these cells (Fig. 5E). In contrast to *ATF4* that uniformly regulated the expression of all post-natal microglia, TATA-box binding protein associated factor 1 (*TAF1*), CCCTC-binding factor (*CTCF*), nuclear factor, interleukin 3 regulated (*NFIL3*) and nuclear factor kappa B subunit 1 (*NFKB1*) regulated the transcriptomic expression of specific microglia populations (Fig. 5F).

Altogether, our analysis revealed that each stage of human microglia development has specific TF content at the expression level and the activity of TFs broadly clusters microglia into pre-natal and post-natal populations. Pre-natal microglia have higher activity of TFs that control cell proliferation while adult specific TFs mainly control the immune signature of microglia.

### Transcriptional comparison of human and mouse microglia across developmental ages

**Fig. 6:**
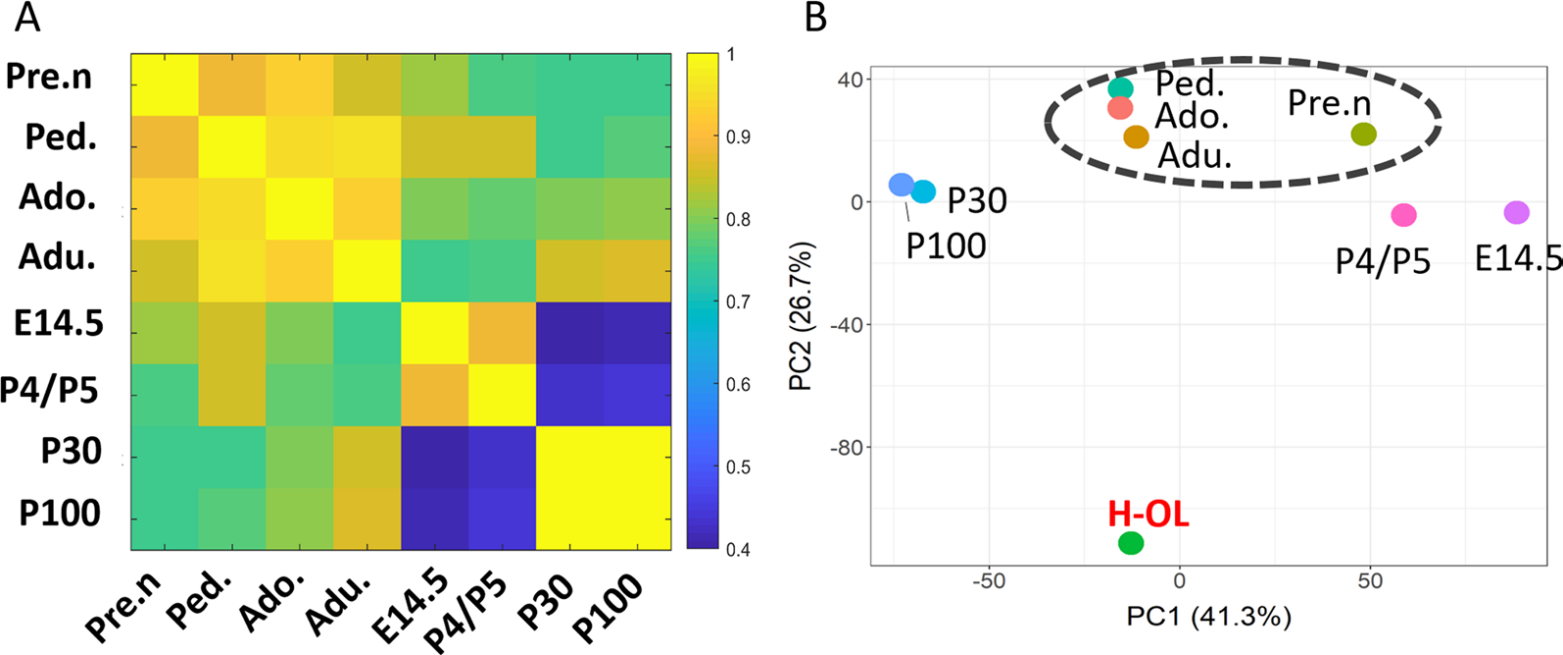
Transcriptional comparison of human and mouse microglia across developmental ages. **A** Heatmap showing Pearson correlation coefficient score of human and mouse microglia at different ages. The score is represented by a color gradient in which yellow is the highest score (100%) and blue is lowest (40%). **B** PCA plot of human and mouse microglia at different ages of development. *Pre n* Pre-natal, *Ped* Pediatric, *Ado* Adolescent, *Adu* Adolescent, *H-OL* Human Oligodendrocyte. The average of three adult human oligodendrocyte samples sequenced at the same time as the microglia (Additional file 1: Fig. S1A, Additional file 5: Table S6) was used to verify the absence of batch effects between samples

To assess the relevance of findings made in the murine system to human microglia biology, we compared our dataset to that of Hammond et al., who studied developmental heterogeneity of mouse microglia at similar timepoints to ours [17]. Hammond et al. used mouse microglia derived from embryonic day (E14.5), early post-natal day 4/5 (P4/P5), a juvenile stage (P30), and adulthood (P100). Even though it is generally accepted that there is no perfect way to correlate age between species we considered the samples provided by Hammond et al. equivalent to those of human pre-natal, pediatric, adolescent and adult timepoints, based on previous publication [40]. Samples were analyzed separately and the average expression of each gene per timepoint was used to measure the correlation between age groups and to perform a principal component analysis (PCA). The average of three adult human oligodendrocyte samples sequenced at the same time as the microglia (Additional file 1: Fig. S1A, Additional file 5: Table S6) was used to verify batch effects between samples. A combat algorithm was then run to remove the batch effect. Samples were found to have a high degree of similarity (correlation coefficient =  ~ 0.8) (Fig. 6A). According to the PCA plot, human oligodendrocytes clustered separately, indicating a lack of batch effect (Fig. 6B). This plot revealed that human pre-natal samples had a distinct expression profile from human post-natal samples. E14.5 and P4/P5 mouse microglia clustered together but remained separate from P30 and P100 microglia (Fig. 6B). These two early developmental stages of mouse microglia also clustered close to human pre-natal cells. These observations suggest that mouse P4/5 microglia have an expression profile similar to human and mouse pre-natal microglia (Fig. 6B). We obsereved the same result upon plotting the samples without the oligodendrocyte samples (Additional file 3: Fig. S3D). Interestingly, human pediatric microglia clustered closely with human adolescent and adult samples. This contrasted with mouse pediatric microglia that clustered separately from mouse adolescent and adult microglia. This observation might suggest that there is a delay in the maturation of mouse pediatric microglia compared to their human counterparts, even though further experimentation is required to prove this hypothesis.

In summary, correlation analysis between human and mouse microglia samples across different stages of development suggests that, despite similarities between human and mouse cells, microglia tend to cluster in a species-specific manner rather than on based on their developmental age.

## Discussion

In our study, we characterized the transcriptomic profile of microglia at a single cell level across pre-natal, pediatric, adolescent, and adult stages of human development. One of our primary objectives was to determine if human microglia evolve transcriptionally over time. Our data suggests that microglia maturation does not follow a pre-determined developmental program, but rather the cells acquire specific functional states likely as a result of changing microenvironments encountered over time. Previously, Kracht et al. studied the development of human pre-natal microglia at gestational week (GW) 9–18 and reported that human pre-natal microglia acquired a mature phenotype by GW12. Microglia continued to develop up until GW18, at which point they were considered to be fully mature [23]. In line with this observation, our pre-natal samples (GW 13–19) were not significantly different from post-natal microglia when subjected to pseudotime trajectory analysis. This result supports the idea that human microglia development starts at an early gestational timepoint and is completed by mid-gestation. Based on our data and that of Kracht et al., the differences between transcriptional states of microglia at various stages of development beyond GW18 appear to be in response to the changes in the surrounding CNS tissue as opposed to the existence of a predetermined microglia developmental program.

Given the significant differences between the pre- and post-natal CNS environment, it was unsurprising to observe that the greatest separation between microglia of different ages came between pre- and post-natal microglia, also previously observed in mouse studies [17, 19]. The strongest suggested functional difference between pre- and post-natal microglia was that of pre-natal cells having a greater phagocytic capacity. The assessment of functional differences between microglia of different ages using in vitro methods is difficult. Due to the scarcity of the human materials, it is difficult to perform experiments on microglia from a wide range of donors side by side. Moreover, significant transcriptional alterations have been shown to occur when microglia are maintained in vitro [41, 42]. For example, the gene encoding MerTK, major regulator of myelin uptake by microglia [43], has been shown to be downregulated in vitro compared to freshly isolated ex vivo microglia [41, 42]. In contrast, CD14 has been shown to be upregulated in vitro [44]. One advantage of using scRNA-seq on freshly isolated microglia is that it allows us to detect differences between age groups that might be otherwise masked by in vitro culture conditions. Evidence from multiple models suggests that microglia display significant phagocytic activity early in brain development that is critical for the proper establishment of the CNS neuronal architecture [45]. This relates to microglia-mediated regulation of neural progenitor cell (NPC) number, phagocytic clearance of synaptic material and the clearance of excess or dead neurons [46, 47]. In line with these evidence and consistent wih our transcriptional observations, we confirmed that *CD36*, a positive regulator of phagocytosis, was highly expressed in human pre-natal brain tissue and minimally expressed (by comparison) in human adult brain tissue in situ, suggesting higher phagoctic activity of the human pre-natal microglia.

A comprehensive comparison of both human and mouse microglia is needed to better translate findings from mouse studies to basic human microglia biology and to understand the contribution of microglia to human disease. It has been shown that adult microglia from ten different species display some differences in their gene expression profile, though the core gene expression program in many species overlaps [21]. Additional studies compared mouse and human microglia but limited their study to specific lists of genes [23]. In our study, we compared the full transcriptional content of human and mouse microglia at specific ages and found a strong correlation between cells of both species at each age. Despite the similarity, human and mouse microglia exhibited a stronger correlation with the other developmental stages of their own species than with the corresponding developmental stage of the other. In addition, we observed a notable difference between human and mouse early post-natal microglia. Hammond et al. showed that mouse P4/P5 microglia have a more similar expression signature to pre-natal cells compared to the P30 and P100 microglia [17]. In contrast, human microglia of an equivalent age were found to be more similar to microglia of other post-natal stages (adolescent and adult) than to pre-natal microglia. This suggests that intrinsic species differences between human and mouse should always be taken into account for translational studies.

In our study, we found that human microglia do not exhibit distinct maturation stages but rather acquire transcriptional signatures in response to a continuously evolving brain microenvironment. Nonetheless, microglia from each age point do display an enhancement of particular transcriptional states that suggest an importance of certain functions. For example, some microglial clusters had higher expression of inflammatory genes like *IL1B* and *MSR1,* which are well established drivers of pro-inflammatory cellular responses [3, 48]. These clusters were heavily enriched within the post-natal cells, specifically adult cells. In addition, several interferon-response genes such as *CCL3* and *CCL4* were increased in adult microglia compared to microglia from younger donors. This observation implies that adult human microglia are more “primed” to respond to infectious or damaging insults. In fact, we confirmed higher secretion of pro-inflammatory cytokines by post-natal cells compared to the pre-natal upon in vitro LPS treatment. In line with this observation we further confirmed higher expression of *IL18* and *CXCR4* mRNA in an adult human brain sample compared to pre-natal. Altogether, our data suggest that post-natal microglia are more immune responsive than pre-natal ones. We also identified microglia that expressed the *MS4A* gene family, which has been introduced by Hammond et al. as a group of cells that express both microglia and macrophages markers [17]. They found this *MS4A* positive cluster of cells at early developmental stages in mice while in contrast, we identified these cells to be enriched in post-natal samples. In line with our data, a recent study suggests that these cells may come from a second wave of microglia development, in which the cells proliferate in the pre-natal liver before seeding the brain tissue [35].

We investigated the transcription factor (TF) expression and activity levels of microglia to supplement our transcriptional analysis. The TF content of microglia at different ages has been documented in mice in two separate studies [16, 49]. In a study of human microglia, Kracht and colleagues studied the TFs underlying the maturation of microglia from early gestational weeks (GWs) to later GWs [23]. They found that gene expression programs switched from general cellular processes to microglia-specific functions during later gestational timepoints (GW 12–18) [23]. In our study, we found that microglia from each age had a moderately unique TF profile at the expression level. The high expression of *SOX4* in pre-natal microglia is consistent with the finding of Kracht et al. in which an upregulation of *SOX4* in the early gestational weeks was reported [23]. Expression of the cell cycle repressor gene *MAFB* is highest at the pediatric age, which is consistent with lower proliferation capacity of post-natal microglia [50]. *CEBPD* is critically involved in the regulation of lipid metabolism pathway of macrophages [51]. Higher expression of this TF during adolescence correlates with our gene ontology finding in which we observed higher metabolic activity of microglia at this age. Finally, *PRDM1* expression was shown to be highest in adult microglia, *PRDM1* has been shown to be involved in polarizing macrophages to a pro-inflammatory phenotype and this correlates with our observation of an enhanced inflammatory signature in adult microglia [52]. At the level of TF activity, pre-natal microglia were most distinct from the post-natal cells. For example, we found *ATF4* to be highly active in the post-natal cells as compared to pre-natal microglia. *ATF4* is known to have a role in positively regulating the inflammatory signature of microglia [38]. This observation suggests a higher inflammatory signature of post-natal microglia and is in agreement with our previous transcriptional findings. Conversely, we found increased activity of *E2F4* in the pre-natal microglia, which has been shown to be a cell cycle regulator [53]. Increased activity of TFs involved in the cell cycle in the pre-natal microglia is in line with the well-documented higher proliferative capacity of microglia at this developmental stage, as seen in rodent models [2]. Finally, *SP1* which has been shown to be involved in chromatin remodeling and was found to display higher activity in pre- versus post-natal microglia and *SOX4* have previously been identified to control general cellular functions at early developmental stages of microglia by Kracht et al. [23].

## Conclusion

Here we provide a comprehensive single cell analysis of the transcriptional profile of human microglia at different ages. The major focus of the existing studies evaluating single cell transcriptional signatures of human microglia has been on cells from pre-natal [23] or adult ages [19, 22], with a complete lack of data on cells from pediatric and adolescent ages. We identified a wide range of microglia transcriptional states during development which were associated with specific functions. Pre-natal cells were found to have a unique gene expression profile compared to post-natal cells, including a signature that suggests greater phagocytic capacity. Conversely, post-natal microglia displayed a continuum of gene expression, with only subtle differences between cells of different ages. Post-natal microglia exhibited a transcriptional signature indicating greater inflammatory capacity compared to pre-natal cells. We believe the distinct signature observed between pre- and post-natal cells is in response to changes in the brain microenvironment and fluctuations in systemic hormonal levels and not due to the existence of a predetermined microglia developmental program. We also explored the GRNs governing the differences between microglia of different ages and found that TF expression levels and activity differed the most between pre- and post-natal microglia. Despite high transcriptional similarity between human and mouse microglia of all age groups, microglia clustered more closely with the cells of their own species than with other cells of the same age. We acknowledge the limitation that due to the source of tissues we were unable to study regional heterogenity of microglia in the brain parenchyma and therefore there is a possibility there might be smaller populations of microglia not assessed in our analysis. Further studies are needed to gain a better understanding of the molecular mechanisms that underlie human microglia development and to understand how the aging process may contribute to the loss of homeostatic microglia functions and/or the acquisition of pathological microglia phenotypes.

## Supplementary Information

**Additional file 1: Figure S1.** Cellular composition of the brain tissue after tissue processing captured by single cell RNA sequencing. A) UMAP plot showing the cell identities captured by single cell RNA sequencing in the post-natal datasets. Mic = Microglia, MOL = mature oligodendrocyte, lOPC = Late oligodendrocyte progenitors cells, eOPC = Early oligodendrocyte progenitors cells, BAM = Border associated macrophage, Ast = Astrocyte, Lymp = Lymphocyte. B) Violin plot indicating average expression of marker genes for each identified cells type. Red rectangles denote the cells that were selected for the downstream analysis. C) Violin plot indicating average expression of PTPRCacross all ages in addition to FACS of microglia sorting using CD11b and CD45 antibodies. D) UMAP plot showing clusters of pre-natal microglia after tissue processing and single cell RNA sequencing and normalized average expression of microglia canonical marker genes and cell proliferation marker genes. E) UMAP plots indicating distribution of 12 individual datasets which were used in our study. F) DotPlot depicting expression of microglia canonical genes across all clusters. G) Violin plot indicating average expression of *MS4A4A* and *MS4A7* genes in cluster 13.

**Additional file 2: Figure S2.** Higher phagpcytic capacity of the pre-natal microglia. A) Pre-natal and post-natal microglia in culture were exposed to pHRodo Green-labelled myelin debris for 15 min, 30 min and 2 h. Cells were then counterstained with Hoechst 33342 and the green fluorescence intensity per cell was measured. The left scatter plot shows the mean green fluorescence intensityafter 2 h. The right scatterplot shows the estimated amount of time after which half of the maximal uptake capacity of the cells is reached. B) Fluorescence images of pre- and post-natal microrglia in after exposure to pHRodo Green-labelled myelin debris for 15 min, 30 min and 2 h and counter counterstained with Hoechst 33342.

**Additional file 3: Figure S3.** Pre-natal microglia have distinct transcription factor activity compared to post-natal cells. A) SCENIC plot depicting unbiased microglia clustering according to inferred TF activity separated by cluster. B) Heatmap showing the activity of 142 identified TFs during SCENIC analysis. The activity score of TFs is represented by color coded z-score. C) SCENIC plot representing mRNA expression and binary regulon activity of *SPI1* TFs inferred by SCENIC. Expression of genes is represented by a color gradient in which orange means high expression and grey means no expression. Activity is represented by a binary index in which blue means active and grey means inactive. D) PCA plot of human and mouse microglia at different ages without human oligodendrocyte samples. Pre.n = Pre-natal, Ped. = Pediatric, Ado. = Adolescent, Adu. = Adolescent, H-OL = Human Oligodendrocyte.

**Additional file 4: Table S1.** Pathology and sex of used brain tissues.

**Additional file 5: Table S2.** The list of up regulated genes in each cluster identified through differential gene expression analysis. **Table S3.** The average expression of genes per developmental stage. **Table S4.** Top 100 highly variable genes per developmental stage. The average expression and standard deviation of genes are represented. **Table S5.** The list of genes whose expression decreased and incteased with age. Average expression of genes are represented. The genes are ordered according their standard deviation across age groups from highest to lowest. **Table S6.** Average expression of mouse and human samples which are used to perform principle component analysis. Average expression of pooled human oligodendrocyte samples is also represented.

## Data Availability

All data needed to evaluate the conclusions in the paper are present in the paper. The raw data of this study is available under the GSE160813 Accession number. The data of one pre-natal FACS sorted sample will be made available upon acceptance of the manuscript. To facilitate the use of our scRNA-seq datasets, we have also developed a straightforward online tool for evaluating gene expression at single-cell resolution (https://stratton-lab.github.io/dataviz) [24].
