# Supplementary figures and images for: Analysis of the microglia transcriptome across the human lifespan using single cell RNA sequencing
Source: J Neuroinflammation. 2023 May 30;20:132. doi: 10.1186/s12974-023-02809-7 (PMC10230780; doi:10.1186/s12974-023-02809-7)

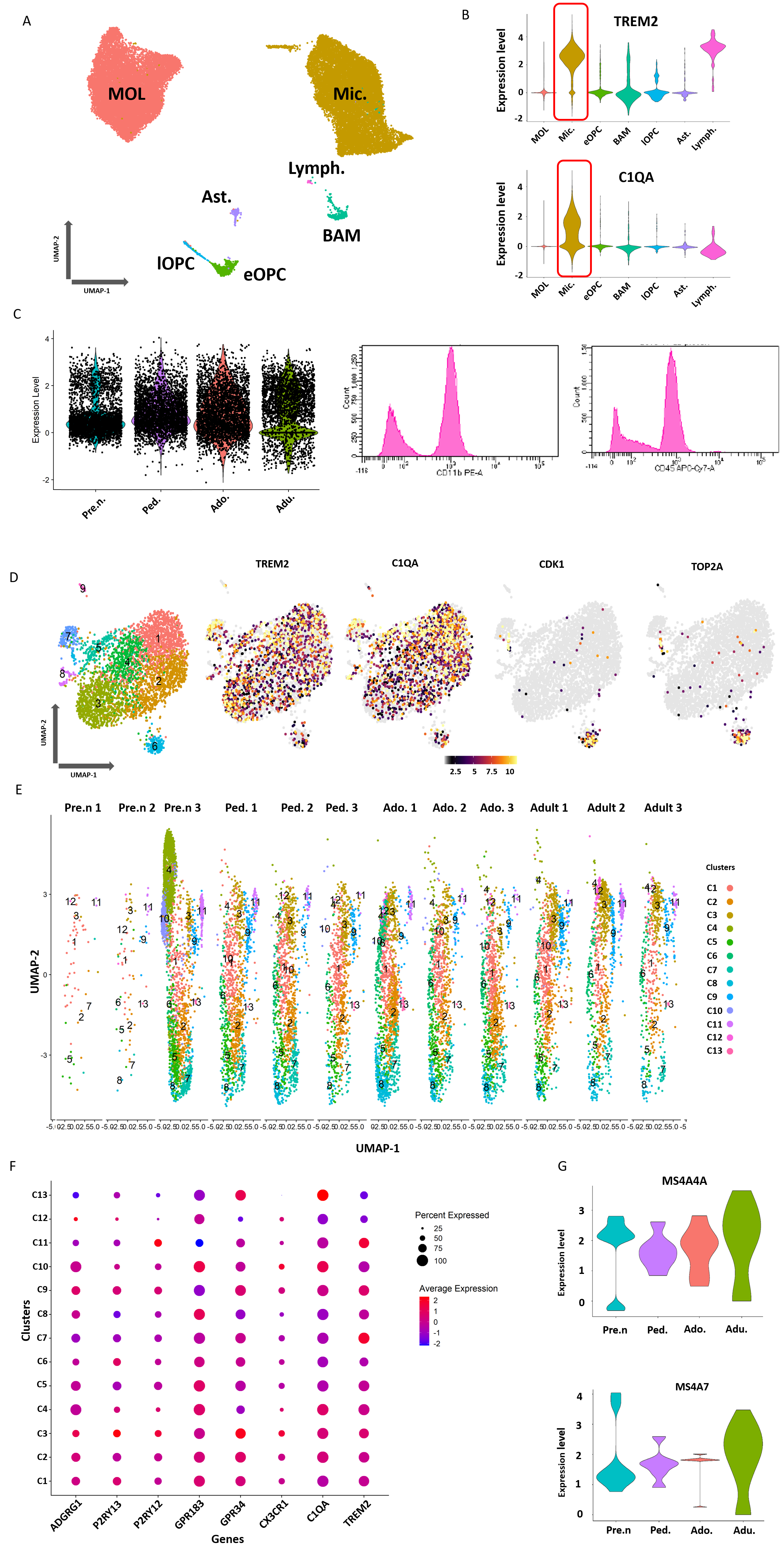

Supplement: Supplementary file 1 — Additional file 1: Figure S1. Cellular composition of the brain tissue after tissue processing captured by single cell RNA sequencing. A) UMAP plot showing the cell identities captured by single cell RNA sequencing in the post-natal datasets. Mic = Microglia, MOL = mature oligodendrocyte, lOPC = Late oligodendrocyte progenitors cells, eOPC = Early oligodendrocyte progenitors cells, BAM = Border associated macrophage, Ast = Astrocyte, Lymp = Lymphocyte. B) Violin plot indicating average expression of marker genes for each identified cells type. Red rectangles denote the cells that were selected for the downstream analysis. C) Violin plot indicating average expression of PTPRCacross all ages in addition to FACS of microglia sorting using CD11b and CD45 antibodies. D) UMAP plot showing clusters of pre-natal microglia after tissue processing and single cell RNA sequencing and normalized average expression of microglia canonical marker genes and cell proliferation marker genes. E) UMAP plots indicating distribution of 12 individual datasets which were used in our study. F) DotPlot depicting expression of microglia canonical genes across all clusters. G) Violin plot indicating average expression of MS4A4A and MS4A7 genes in cluster 13. [file 12974_2023_2809_MOESM1_ESM.png]

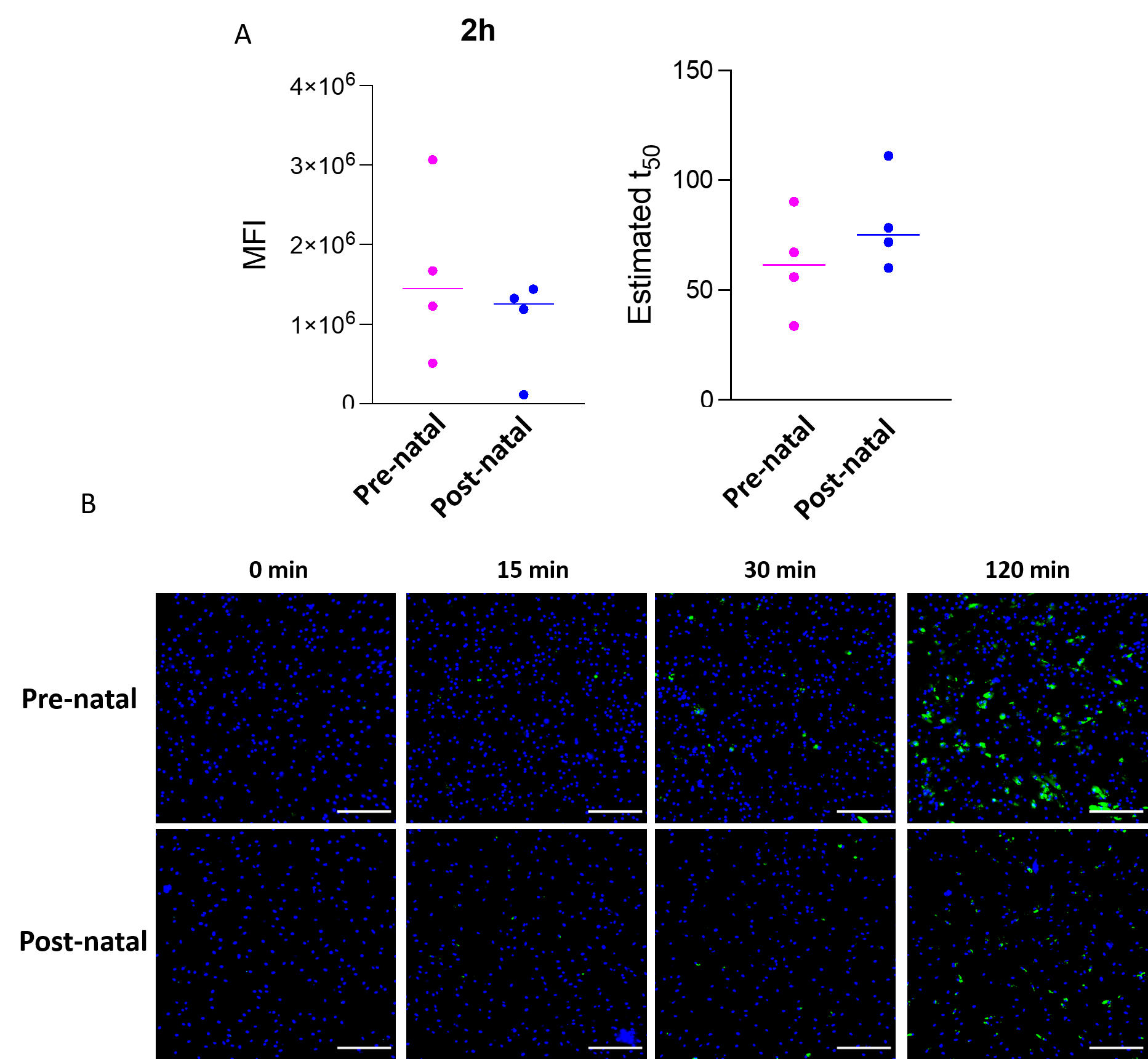

Supplement: Supplementary file 2 — Additional file 2: Figure S2. Higher phagpcytic capacity of the pre-natal microglia. A) Pre-natal and post-natal microglia in culture were exposed to pHRodo Green-labelled myelin debris for 15 min, 30 min and 2 h. Cells were then counterstained with Hoechst 33342 and the green fluorescence intensity per cell was measured. The left scatter plot shows the mean green fluorescence intensityafter 2 h. The right scatterplot shows the estimated amount of time after which half of the maximal uptake capacity of the cells is reached. B) Fluorescence images of pre- and post-natal microrglia in after exposure to pHRodo Green-labelled myelin debris for 15 min, 30 min and 2 h and counter counterstained with Hoechst 33342. [file 12974_2023_2809_MOESM2_ESM.png]

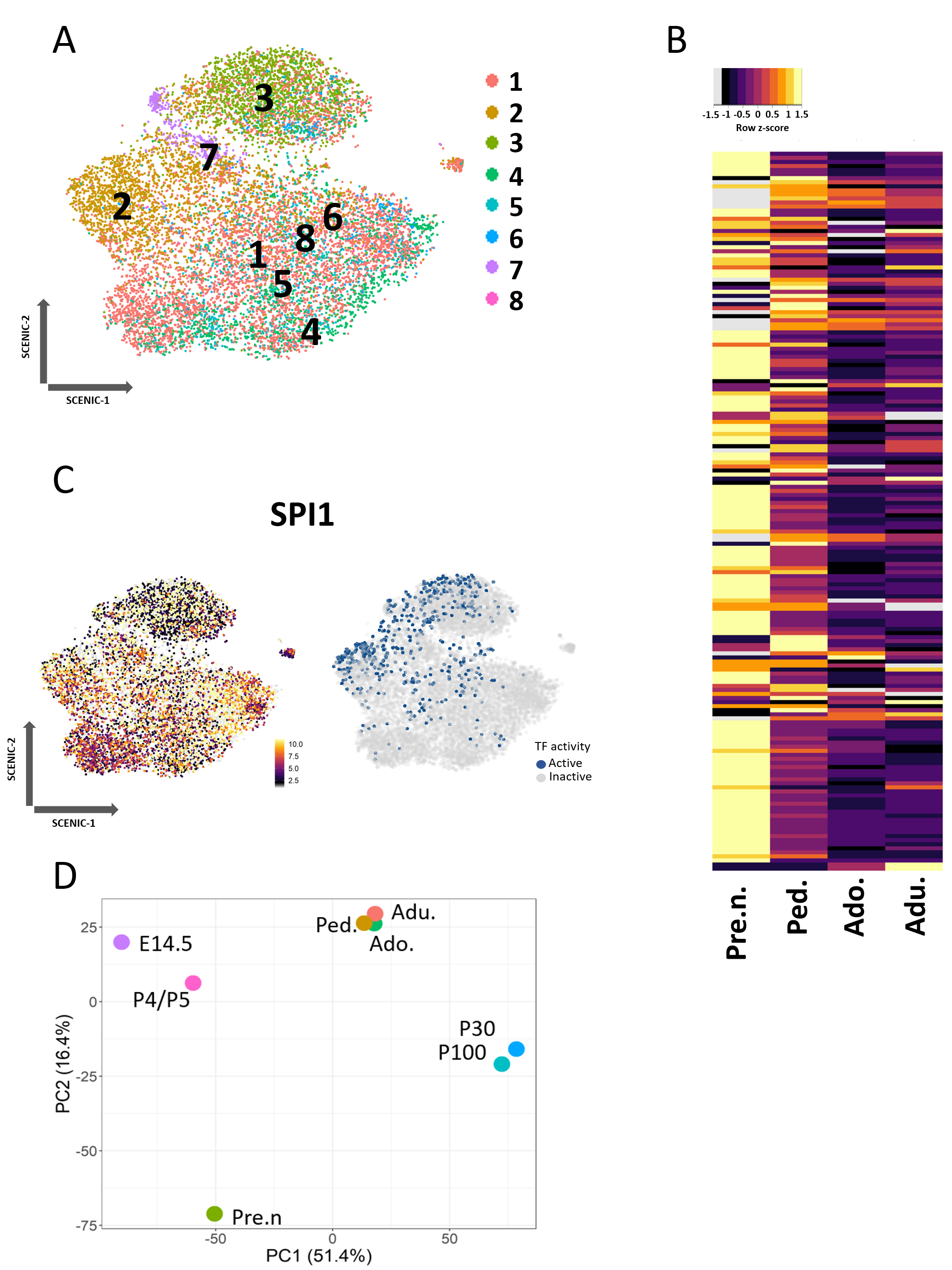

Supplement: Supplementary file 3 — Additional file 3: Figure S3. Pre-natal microglia have distinct transcription factor activity compared to post-natal cells. A) SCENIC plot depicting unbiased microglia clustering according to inferred TF activity separated by cluster. B) Heatmap showing the activity of 142 identified TFs during SCENIC analysis. The activity score of TFs is represented by color coded z-score. C) SCENIC plot representing mRNA expression and binary regulon activity of SPI1 TFs inferred by SCENIC. Expression of genes is represented by a color gradient in which orange means high expression and grey means no expression. Activity is represented by a binary index in which blue means active and grey means inactive. D) PCA plot of human and mouse microglia at different ages without human oligodendrocyte samples. Pre.n = Pre-natal, Ped. = Pediatric, Ado. = Adolescent, Adu. = Adolescent, H-OL = Human Oligodendrocyte. [file 12974_2023_2809_MOESM3_ESM.png]
