# Supplementary material for: Analysis of the microglia transcriptome across the human lifespan using single cell RNA sequencing
Source: J Neuroinflammation. 2023 May 30;20:132. doi: 10.1186/s12974-023-02809-7 (PMC10230780; doi:10.1186/s12974-023-02809-7)
Supplement: Supplementary file 4 — Additional file 4: Table S1. Pathology and sex of used brain tissues. [file 12974_2023_2809_MOESM4_ESM.docx]

| **Sample** | **Age** | **Sex** | **Diagnosis** |
| --- | --- | --- | --- |
| Fetal 1 | G.W. 13 | M | - |
| Fetal 2 | G.W. 18 | M | - |
| Fetal 3 | G.W. 19 | F | - |
| Pediatric 1 | 1.5 | M | Focal cortical dysplasia |
| Pediatric 2 | 2 | M | Chronic encephalitis; (Rasmussen) post-surgical encephalomalacia |
| Pediatric 3 | 2 | F | Focal cortical dysplasia; polymicrogyria |
| Adolescent 1 | 10 | F | Focal cortical dysplasia IIa,b |
| Adolescent 2 | 13 | F | No pathologic diagnosis |
| Adolescent 3 | 14 | F | Focal cortical dysplasia IIId |
| Adult 1 | 41 | F | Grey/white matter gliosis |
| Adult 2 | 51 | F | Mesial temporal sclerosis |
| Adult 3 | 62 | M | Mesial temporal sclerosis |

Supplementary table 1- Demographics of the patients used in this study.
